# Genomic analysis of extended-spectrum beta-lactamase-producing *E. coli* from Czech diary calves and their caretakers

**DOI:** 10.3389/fvets.2025.1552297

**Published:** 2025-03-12

**Authors:** Martina Masarikova, Aneta Papouskova, Iva Sukkar, Jarmila Lausova, Darina Cejkova, Alois Cizek

**Affiliations:** ^1^Department of Infectious Diseases and Microbiology, Faculty of Veterinary Medicine, University of Veterinary Sciences Brno, Brno, Czechia; ^2^CEITEC VETUNI Brno, University of Veterinary Sciences Brno, Brno, Czechia; ^3^Department of Microbiology, Faculty of Medicine in Pilsen, Charles University, Pilsen, Czechia; ^4^Department of Biomedical Engineering, Faculty of Electrical Engineering and Communication, Brno University of Technology, Brno, Czechia

**Keywords:** *Escherichia coli*, ESBL, dairy cattle, zoonotic transmission, antimicrobial resistance, whole-genome sequencing

## Abstract

**Introduction:**

The increasing prevalence of antimicrobial resistance in livestock, particularly the dissemination of extended-spectrum beta-lactamase-producing *Escherichia coli*, poses a significant zoonotic and public health risk. This study investigates the genomic characteristics of cefotaxime-resistant *E. coli* isolates from dairy calves across 23 Czech farms and their caretakers.

**Materials and methods:**

Bacteriological cultivation on McConkey agar with cefotaxime was used for their isolation, susceptibility to selected antibiotics was determined by disc diffusion method, production of extended-spectrum beta-lactamases (ESBL) was demonstrated by double disc synergy test. The PCR was applied to confirm the presence of selected genes encoding resistance to some beta-lactams and genes encoding resistance to quinolones carried on plasmids. Using whole-genome sequencing, we evaluated resistance genotypes, sequence types, serotypes, plasmid replicons, and virulence genes.

**Results and discussion:**

Among 266 rectal samples obtained from the calves, 128 (48%) harbored cefotaxime-resistant *E. coli*. Whole-genome analysis revealed *bla_CTX-M_* genes in 91% (116/128) of isolates, with *bla*_*CTX-M*-14_ (44%) and *bla*_*CTX-M*-1_ (34%) being the dominant variants. Other beta-lactamase gene *bla_TEM-1b_* was found in 40% (51/128) of isolates. Notably, no cephamycin resistance genes have been identified. The plasmid-mediated quinolone resistance (PMQR) gene *qnrS1* was present at 21% (27/128) of isolates. The colistin resistance gene *mcr-1* was found in a single ST2325 isolate. Sequence typing revealed significant clonal diversity, with 21 different STs detected among 68 sequenced isolates. ST10 was the most prevalent (27%), followed by ST69 (12%), ST29 (7%) and others. The phylogenetic distribution showed a predominance of commensal groups A (54%) and B1 (21%). The most common serotypes included O101:H9 (21%), O15:H18 (12%), H12, and O70:H11 (7%). Analysis of plasmid content revealed a complex distribution of 18 distinct plasmid replicon types, especially IncF, followed by Col-type and IncI1-type plasmids. Cross-species transmission was indicated by the detection of clonal strains shared between calves and caretakers, notably ST10-O101:H9 and ST34-O68:H30. The prevalence of high-risk clones and the presence of mobile resistance elements underscore the urgent need for stringent monitoring, antimicrobial stewardship, and improved biosecurity measures in livestock environments like increased caution and personal hygiene of animal handlers to mitigate the spread of resistant *E. coli* between animals and humans.

## 1 Introduction

Fecal carriage of Enterobacteriaceae resistant to the most critical antimicrobials including 3rd and 4th generation cephalosporins is frequently reported in livestock animals, including dairy cattle (1, 2). Albeit most of the extended-spectrum beta-lactamases (ESBL) producing strains are commensals, they serve as a reservoir of antimicrobial resistance (AMR) genes and the associated genetic elements (most importantly plasmids). Livestock may represent an environment for selection of potentially zoonotic resistant strains, the risk of which is promoted by antimicrobial use. Several studies assessed potential risk factors promoting ESBL/AmpC-producing *E. coli* presence and spread, concluding that in general dairy cattle is at higher risk compared to beef cattle, which could be attributed to differences in management, housing and probability of antimicrobial treatment (2–5). While introduction of high-risk clones (e.g., ST131) from animals to humans via food chain remains a hypothesis and the frequencies of different sequence types and resistance genes differ between animal and human populations (6), contact transmission between animals and farm staff takes places commonly, as has been repeatedly reported (7–9, 37). Recent research suggests there are differences between phylogenetic groups and sequence types in their ability to spread and adapt to different hosts and environments; some strains may colonize both humans and animals more readily, as well as, e.g., form biofilms and/or gain resistance/virulence-associated mobile genetic elements (10). Understanding the specific mechanisms underlying transmission and persistence is critical to introduction of efficient pathogen-focused control measures. Besides ESBL-producing *E. coli*, cattle are a common asymptomatic carrier of Shiga toxin-producing *E. coli* (STEC), which can be transmitted to humans both by a direct contact and food contamination (11). In this study, we performed a genomic analysis of cefotaxime-resistant *E. coli* isolates obtained from calves originating from multiple Czech dairy farms to evaluate the prevalence of sequence types (STs), serotypes, replicon types, resistance and virulence genes and the occurrence of genotypes with suspected zoonotic potential, along with the assessment of potential risk factors associated with ESBL fecal carriage. Furthermore, *E. coli* isolates obtained from three workers from the farm, where the sampling took place, were also included in the study.

## 2 Materials and methods

### 2.1 Sample collection

**Figure 1:**
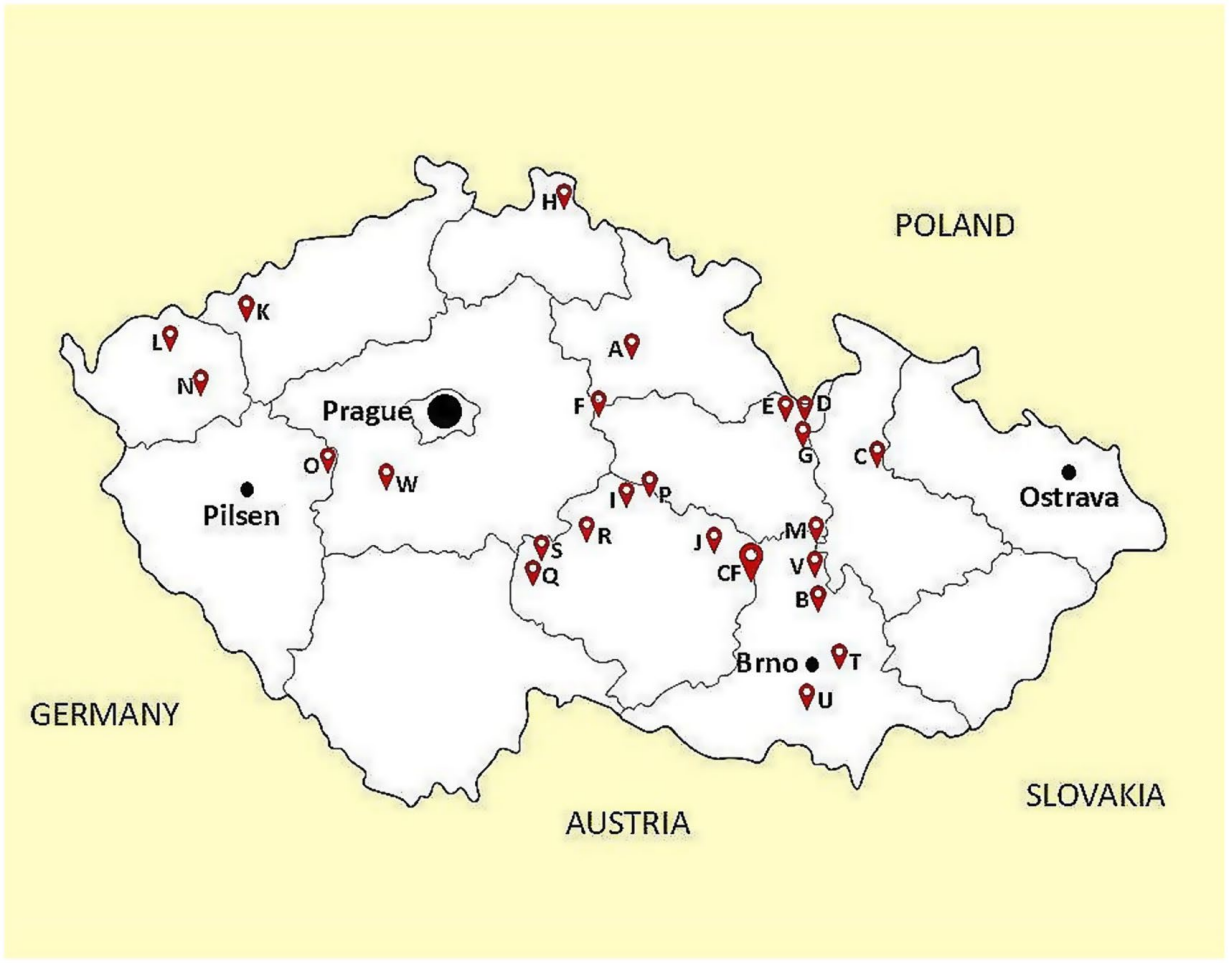
The map of the Czech Republic with localization of sampled farms.

The rectal swab samples from calves were obtained from 23 different dairy farms (A–W) across various regions of the Czech Republic from August to October 2019 (see Figure 1). This sampling was carried out as part of the routine health examination of the newly admitted animals by the supervising veterinarian according to the established methodology. Calves from farms A–R and W were transported to a collection farm (CF) where is a continual flow of animals, i.e., calves of different origin are housed together to form groups about 20 individuals. Rectal swab samples from calves were there taken at the arrival before formation of these groups to minimize the possibility of cross-contamination. Samples from farms S, T, U and V were delivered separately by veterinarians directly from the farms of origin. A total of 266 animal samples were obtained, one sample represented one calf. In addition, rectal swabs were sampled from three caretakers and one veterinarian working at the collection farm. All human subjects provided written informed consent and performed self-sampling.

### 2.2 *Escherichia coli* isolation

The collected material was enriched in buffered peptone water (Oxoid, United Kingdom) under aerobic conditions for 24 h at 37°C, 10 μL of each enriched peptone waters were then cultured for cefotaxime-resistant *E. coli* on McConkey agar supplemented with cefotaxime (2 mg.L^−1^) (MCA_cef_) (Sigma-Aldrich, Germany). Inoculated plates were cultured under aerobic conditions for 48 h at 37°C and presumptive lactose-positive colonies with characteristic morphology were identified by MALDI-TOF MS method using the Microflex LT instrument equipped with MALDI Biotyper software version 3.1 and the MALDI Biotyper^®^ library version MBT 6903 MSP (Bruker Daltonics, Germany). Isolates were classified as *E. coli* if the identification score was greater than 2.0. The colonies definitely confirmed as *E. coli* were subcultured on McConkey agar without cefotaxime and finally on Columbia blood agar (Oxoid, United Kingdom) and preserved at −80°C in 0.5 mL of cryoprotective medium containing bacteriological peptone and glycerol. If positive, one *E. coli* isolate represented one calf. The human samples were processed analogically, but up to five isolates from each culture were taken, if the culture was positive.

### 2.3 Antimicrobial susceptibility testing and the detection of antimicrobial resistance determinants

The susceptibility to 13 antibiotics, including amoxicillin-clavulanic acid (30 mg), ampicillin (10 mg), cephalotin (30 μg), cefoxitin (30 μg), ceftazidime (10 μg), chloramphenicol (30 μg), ciprofloxacin (5 μg), gentamicin (10 μg), nalidixic acid (30 μg), streptomycin (10 μg), sulfamethoxazole-trimethoprim (25 μg), sulfonamides compound (300 μg), and tetracycline (30 μg) (Oxoid, United Kingdom) was determined by disc diffusion test (DDT). Double-disc synergy test was used to evaluate the production of ESBL and/or AmpC enzymes. Both methods were performed and results were interpreted according to CLSI document (12).

All cefotaxime-resistant isolates obtained from calves and humans were examined via polymerase chain reaction (PCR) for the presence of selected genes encoding beta-lactamases production (*bla*_TEM_, *bla*_SHV_, *bla*_OXA,_ *bla*_PSE,_ *bla*_CTX-M_) and for plasmid-mediated quinolone resistance (PMQR) genes [*qnrA*, *qnrB*, *qnrC*, *qnrD*, *qnrS*, *aac(6′)-Ib-cr*, *qepA*, *oqxA*, *oqxB*]. A list of all primers used in the study is provided by Masarikova et al. (13).

### 2.4 Whole-genome sequencing, assembly, and data analysis

Of 128 isolates obtained from calves, 68 were selected for whole genome sequencing (WGS). On farms where multiple cefotaxime-resistant *E. coli* were isolated, the main selection criterion for WGS was the difference in resistance phenotype, so that the range of isolates tested was as wide as possible. In addition, in some selected *E. coli* we failed to obtain sufficient DNA concentration presumably due to heavy mucoid growth. This phenomenon may be significant and warrants further investigation, as specific genotypes characterized by, for example, an increased ability to persist in the environment or colonize cattle, may have been omitted from the selection. All human cefotaxime-resistant *E. coli* (15 from 3 caretakers) were sequenced.

DNA was extracted using the NucleoSpin Tissue Kit (Macherey-Nagel, Germany) and subjected to short-read sequencing. Sequencing libraries were prepared using the Nextera XT DNA Library Preparation Kit (Illumina, Inc., United States) and sequenced on NovaSeq 6000 platform (Illumina) at UTS Core Sequencing Facility Ithree Institute Australia. Raw Illumina paired end reads were quality (Q ≥ 20) and adaptor trimmed via Trimmomatic v0.39 (14) and assembled via the *de novo* assembler SPAdes v3.13.1 (15).

Genomic sequences were visualized using ResFinder 4.1 (16, 17) for the content of antimicrobial resistance genes, PlasmidFinder 2.1 (18) for plasmid replicons and the virulence factor database (19) was used to assign virulence associated genes. Cut-off for positive detection of tested genes was set up for at least 90% identity and coverage to reference sequences. Sequence type of isolates was determined using MLST 2.0 tool (20), serotypes via SeroTypeFinder 2.0 tool (21) and strain phylotyping was determined using the method described by (22).

Sequence annotations were generated using Prokka v1.14.1 (23) and the annotated assemblies were used for alignment using PIRATE v1.0.4 (24). Maximum-likelihood tree was built using RAxML v8.2.12 (25) with the general time-reversible (GTR) model supported by 500 bootstraps. The single nucleotide polymorphisms (SNPs) matrices between isolates were calculated by snp-dists.^1^ The phylogenetic tree was visualized via iTOL v6 (26, 27). The data are now available under BioProject ID PRJNA1197128.

## 3 Results

### 3.1 Cefotaxime-resistant *E. coli* isolation

A collection of 128 (48%) cefotaxime-resistant *E. coli* was obtained from 266 calves rectal samples originating from Czech dairy farms using selective cultivation; 17 out of 23 sampled farms were positive at least for one *E. coli* strain growing on MCA_cef_ (for an overview of farm positivity/negativity, see Supplementary Table S1). Among human isolates (3 caretakers, 1 veterinarian), only samples from caretakers were positive for *E. coli* isolated via MCA_cef_ (15 human isolates).

### 3.2 Phenotyping and genotyping of cefotaxime-resistant *E. coli*

All isolates obtained from calves showed resistance in disc diffusion test to between two and twelve antibiotics, with 90% being multidrug-resistant (MDR) (resistant to at least three different classes of antibiotics) (115/128). All 128 isolates were resistant to ampicillin, and 127 *E. coli* were also resistant to the first-generation cephalosporin, cephalotin, based on the isolation method using media supplemented with cefotaxime. Resistance to other tested antimicrobials was as follows: tetracycline (73%), streptomycin (70%), sulfonamides compound (59%), chloramphenicol (42%), amoxicillin-clavulanic acid (38%), cefoxitin (36%), ceftazidime (31%), sulfamethoxazole-trimethoprim (30%), nalidixic acid (19%), ciprofloxacin (17%) and gentamicin (13%). Most *E. coli* produced ESBL enzymes (117/128; 91%), 36% produced AmpC (46/128) and 30% (38/128) produced both types of beta-lactamases.

The PCR analysis revealed that 91% of animal isolates harbored *bla*_CTX-M_ (116/128) and 48% carried *bla*_TEM_ genes (62/128). The *qnrS* gene, associated with plasmid-mediated quinolone resistance (PMQR), was found in 21% of *E. coli* isolates (27/128). The results of phenotyping and genotyping of selected resistance genes of all 128 isolates from calves and 15 human *E. coli* are shown in Supplementary Table S2.

### 3.3 Whole-genome sequencing of cefotaxime-resistant *E. coli*: STs, phylogenetic groups and serotypes

Among 68 sequenced *E. coli* strains isolated from calves, 21 various STs were identified. *E. coli* ST10 (27%) and ST69 (12%) predominated, followed by ST29 (7%), ST88 (7%), ST34 (6%), ST56 (6%), ST21 (4%), ST685 (4%), ST2325 (4%) ST48 (3%), ST746 (3%), and ST1202 (3%). Only one isolate was detected for ST38, ST58, ST117, ST155, ST657, ST947, ST1433, ST3381, and ST5635.

The strains from calves were assigned to six different phylogenetic groups, A (37/68, 54%), B1 (14/68, 21%), C (5/68, 7%), D (9/68, 13%), E (1/68, 2%), and G (2/68, 3%).

A total of 27 diverse serotypes were identified among the calf *E. coli* isolates, with O101:H9 (21%), O15:H18 (12%), H12 and O70:H11 (7%) being the most prevalent, other serotypes were represented by one to three isolates (for the results of WGS analysis of 68 animal and 15 human isolates, see Supplementary Table S3).

### 3.4 Detection of AMR genes and plasmids by WGS

Genomic analysis of 68 animal *E. coli* confirmed the presence of *bla*_CTX-M_ type beta-lactamase gene in 67 isolates. The most frequently detected beta-lactamase gene in animal isolates was *bla*_CTX-M-14_ (30/68; 44%), followed by *bla*_CTX-M-1_ (23/68; 34%). The *bla*_CTX-M-8_ type was detected in three isolates (4%), and the *bla*_CTX-M-15_ type was detected in 11 isolates (16%), with a strong association to ST69 lineage in eight cases. Additionally, 27 isolates (40%) also carried the gene for beta-lactamase *bla*_TEM-1b_.

Aminoglykoside resistance genes were commonly detected: *strA*/*B* (42/68; 62%), *aad1* (19/68; 28%), *aad2* (18/68; 27%), *aph*(3′)-Ia (13/68; 19%). For amphenicols, the following genes were detected: *floR* (26/68; 38%) and *cmlA1* (15/68; 22%). Sulfonamide resistance was associated with *sul2* (47/68; 39%), *sul1* (6/68, 9%) and *sul3* (2/68; 3%). Trimethoprim resistance genes detected included *dfrA12* (16; 24%) and *dfrA1* (15; 22%). Tetracycline resistance was associated with *tet*(A) (49; 72%), *tet*(M) (13; 19%) and *tet*(B) (5, 7%). The PMQR *qnrS1* gene was detected in 12 isolates, all of which belonged to phylogenetic group D (ST69, ST38). The *mcr-1* gene for colistin resistance was detected in one isolate (Supplementary Table S3).

In human isolates, the ST10 and ST1202 clones (from caretakers Os L and Os K) carried *bla*_CTX-M-14_, while ST34 (from caretaker Os S) carried *bla*_CTX-M-8_. These strains were multi-drug resistant, carrying a broad range of genes conferring resistance to beta-lactams, aminoglycosides, amphenicols, sulfonamide-trimethoprim and tetracycline resistance. The genes detected included *bla*_TEM-1_, *strA*, *strB*, *aac(3)-IIa, aac(3)-IId, aadA1, aadA2, aph(3′)-Ic, cmlA1, floR*, *sul1*, *sul2*, *dfrA12*, *tet*(A) and *tet*(B). Interestingly, four isolates from the caretaker Os K (Os K e1, e2, e4, e5) harbored also *lnu*(F) gene for lincomycin resistance, which was not detected in any other isolate (Supplementary Table S3).

Altogether 18 different plasmid replicons were detected in the 68 sequenced calf isolates. Each isolate carried at least one plasmid replicon, with a maximum of seven different plasmids and a median of three replicons per isolate. Plasmids of the IncF group were most common, with FIB (62/68; 75%), FII (42/68; 51%), FIA and FIC (both 17/68; 21%). Additionally, plasmids of the Col type (Col4401, ColMG828, pHAD28, Col156, ColRNAI, BS512) were detected in 34 isolates. The IncI1 plasmid was present in 40% of isolates (33 isolates), IncY plasmids in 19% (16 isolates), and IncB/O/K/Z plasmids in 13% (11 isolates). Less common plasmid replicons included IncN (5 isolates), IncHI1 (3 isolates), IncX4 (2 isolates), and IncI2 (1 isolate). Details of the resistance genes and plasmid replicons detected in isolates from calves and their caretakers are given in Supplementary Table S3.

### 3.5 Detection of virulence genes by WGS

Using the available databases, a variety of genes associated with virulence were detected in all 83 (human and animal together) sequenced *E. coli* isolates (Supplementary Table S3). As expected, isolates from phylogenetic group A had the fewest virulence genes. On the contrary, isolates from ST21 and ST29 in phylogenetic group B1 and ST117 from group G carried the most virulence genes. The majority of isolates carried following genes: *hlyE* (83/83, 100%), *gad* (77/83, 93%), *fimH* (71/83; 86%), *csgA* (68/83; 92%) and *traT* (50/83; 60%). The *iss* gene (40/83; 48%) was less common in group A but was present in most isolates from other groups. Some isolates exhibited specific virulence genotype, aligning partially or entirely with defined virotypes. Isolate ST657 could be classified as STEC due to the simultaneous presence of *stx1* and *stx2* while the *eae* gene is absent. Two ST21-O26:H11 isolates carried *stx1/2, astA, ehxA, toxB* + *eae, tir* and other locus of enterocyte effacement (LEE)-associated genes, suggesting the virulence potential, although they cannot be assigned as enterohaemorrhagic *E. coli* (EHEC) solely on the genotypic basis, while another ST21-O26:H11 isolate was *stx* negative but LEE-positive and corresponds to atypical enteropathogenic (aEPEC) (39), as well as five ST29-O70:H11 isolates. The isolates ST117-O24:H4, ST69-O15:H18, ST88-H12, ST947-O32:H25, and ST58-O45:H31 exhibited specific profiles, each carrying varying numbers of genes associated with extraintestinal pathogenicity, such as *iuc/iut, irp2 /fyuA, iroN, cdt, papA, vat, pic, neuC*.

### 3.6 Population structure of cefotaxime-resistant *E. coli*

In the entire set of sequenced isolates, the genomic variability ranged between 0 and 84,186 single nucleotide variants (SNV). The phylogenetic tree revealed several clusters of related isolates, where the genetic distance did not exceed 18 SNVs. Eight ST69-O15:H18-D isolates (from farm A, isolates no. 49, 60, 61, 67, 70, 66, 64, 62) and five ST88:O-:H12-C isolates (from farm S, isolates no. 238, 253, 255, 236, 237) formed separate clusters with genetic distances 0 SNV and 0–4 SNVs, respectively. In both cases, these strains represented the only isolates obtained from these farms.

**Figure 2:**
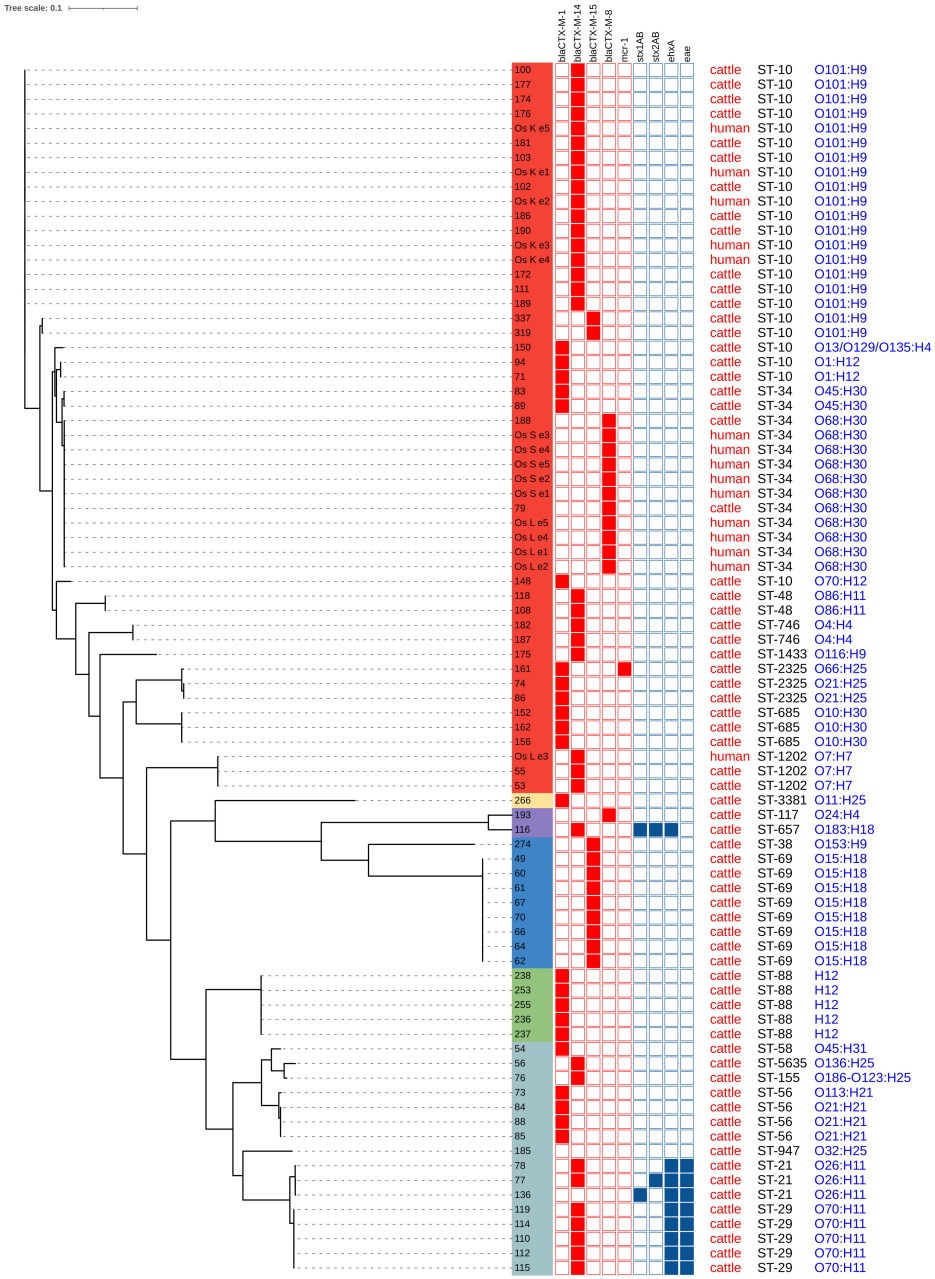
Phylogenetic analysis and characterization of 83 *E. coli* isolated from calves and caretakers. Phylogenic groups are indicated in colors: group A (red), group E (yellow), group G (purple), group D (blue), group C (green) and group B1 (light blue).

For the other farms, where at least four isolates were sequenced, 3–5 different STs were always detected. A cluster of five ST29 isolates (with a variability of 1–17 SNVs) was identified in calves from farms I (isolates no. 119, 114, 110, 115) and J (isolate no. 112). Among the most abundant STs, clusters of 17 isolates (ST10-O101:H9) and 11 isolates (ST34-O68:H30) were prominent, with variability of 0–18 SNVs and 0–8 SNVs, respectively. ST10-O101:H9 isolates were obtained from calves from three different farms (farm J, isolates no. 190, 111, 189; farm P, isolates no. 177, 174, 176, 181, 186, 172; and farm Q, isolates no. 100, 103, 102) and one caretaker (isolates no. Os K e1 - e5). Isolates ST34-O68:H30 originated from two calves from different farms (farm F, isolate no. 79 and farm P, isolate no.188) and two caretakers (isolates no. Os S e1 - e5 and isolates no. Os L e1 - e4). Additionally, one isolate from the second caretaker belonged to a different strain ST1202-O7:H7, which was also detected in two calves (farm C, isolates no. 53 and 55) with 1 SNV variability (for the full population structure, see Figure 2).

Upon closer inspection, clone ST10-O101:H9 was first isolated from calves from farm P on the 15^th^ of August 2019, and from caretaker Os K on the same day. However, this clone subsequently appeared in calves from farms J and P nearly a month later. Two clonally distinct ST10-O101:H9 isolates were observed in October 2019 in calves sampled outside the collection farm. A similar trend was noted for ST34-O68-H30: it was first identified in one calf from farm F on the 14th of August 2019, then the following day in caretakers Os L and Os S, and a month later (6th of September 2019) in a calf from farm P (the dates of samplings are provided in Supplementary Table S3).

## 4 Discussion

Cattle represent the farm animals with the highest prevalence of ESBL/AmpC-producing *E. coli*. Dairy cattle are more at risk than beef cattle, probably due to a different breeding method, where they are more exposed to various risk factors (4, 28). Livestock may pose a risk to humans as a direct source of risk strains, as well as a reservoir of mobile genetic elements with resistance genes. Current research suggests that the diversity of bacterial strains and their genetic elements is fundamentally different in human and animal populations, and effective transmission occurs more or less exclusively from animals to people who are in regular direct contact with them (6, 7).

In our collection, the highest prevalence of beta-lactamase CTX-M-14 was recorded, followed by CTX-M-1 and CTX-M-15. In livestock in Europe, the highest prevalence of type CTX-M-1 is usually reported, followed by CTX-M-15 and 14 (29). In our case, the occurrence of CTX-M-15 was exclusively associated with ST69, while CTX-M-1 was distributed across different sequence types. It is likely that several specific pandemic clones, primarily ST131, are mainly responsible for the global spread of CTX-M-15 (30).

In our collection, no other type of ESBL/AmpC beta-lactamases than CTX-M, e.g., SHV-12, TEM-52 or CMY-2, were represented, although plasmid AmpC beta-lactamases CMY-2 are increasingly reported in humans and livestock in Europe (30). Manga et al. (31) even detected CMY-2 in 94% of cefotaxime-resistant isolates in calves on a dairy farm.

There was a disturbing finding of the *mcr*-1 gene, a plasmid-borne colistin resistance gene, in isolate ST2325 (group A). Despite the use of short-read data, an association with an IncI2-type plasmid could be inferred, which was also described in ESBL-positive STEC isolates from pigs in China (32). Colistin resistance appears to be still relatively low in livestock in Europe, but the occurrence of *mcr*-1 and other variants associated with different types of plasmids is occasionally reported in cattle, pigs and poultry (33, 34, 40).

The predominance of “commensal” phylogenetic groups A and B1 among bovine fecal isolates was expected, although the predominance of one group or the other may vary by study; also the occurrence of phylogenetic groups E, C, D, and G is common, although in a lower prevalence (5, 31, 33, 35). The absence of phylogenetic group B2 among commensal isolates of cattle is typical, although it represents a significant proportion of the commensal microflora in humans. Arimizu et al. (36) in their comparative genomic analysis report a higher adaptation of group B1 to the bovine gut, while group A is more shared between cattle and humans. The strains that are part of the ST10, ST58, ST88, and ST117 complexes are considered to be emergent ExPEC (extraintestinal *E. coli*) lineages, with ST88 and ST117 more closely related to poultry, ST58 more closely related to cattle, and ST10 showing the highest overlap between human and animal isolates (37).

Comparison of our data with other studies shows that the occurrence of different genotypes can be highly variable, and in many cases a particular clone of cefotaxime-resistant *E. coli* dominates on a certain farm, sometimes even persisting for a long time (5, 35), in our case it was farm A (identical clone ST69 in eight calves) and farm S (five ST88 isolates). On the contrary, a diversity of three or more different STs was captured on some farms. Although most of the samples were collected at the collection farm, the time from arrival to collection was very short, and mutual contact and colonization of calves from different farms was unlikely, although not excluded. In contrast, the sampled caretakers were in longer and more intensive contact with all calves and therefore at high risk of colonization.

Two clones, ST10-O101:H9 and ST34-O68:H30, were isolated from calves across several different farms and simultaneously from caretaker Os K and caretaker Os S. In caretaker Os L, along with ST10-O101:H9, clone ST1202-O7:H7, also shared with calves, was detected. It is likely that these findings have an epidemiological connection, given the time coincidence of the detection of these clones in calves and caretakers. It is surprising that both clones were also detected during the next sampling almost a month later. If there was transmission from the calves to the caretaker in the first term, contact must have happened within 24 h. Another possibility is that the caretakers had been already colonized earlier; the continual service in the collection farm may be important, calves from the same farms could have been delivered to the collection farm shortly before. The reverse transmission from the caretakers to the calves cannot be ruled out either. These data do not allow us to draw conclusions about the possible transfer of clones between farms but indicate their epidemic and zoonotic potential. We hypothesize that the clones are in the production chain on an undefinedly long-term basis and are repeatedly introduced to the collection farm where the transmission can occur in both directions. This should be addressed in future with another study. This would require different sampling strategy with more human samples and a detailed history of transmissions and contacts. In the study by Massé et al. (35), this clonal line ST10-O101:H9 was captured from different farms in the sampled area (38) described the clonal transmission of this lineage in a Chinese farm and found a probable association with gulls (Australian silver gull), which figured as a vector. (41) isolated this lineage (with CTX-M-8) from synanthropic pigeons.

A remarkable finding in our study was the capture of isolates ST29-O70:H11 and ST21-O26:H11 producing ESBLs and at the same time representing potential intestinal pathogens like STEC or atypical EPEC. Serotype O26:H11 represents the second most important EHEC serotype and has several sublineages, including ST21 and ST29 (41). Our two ST21-O26:H11 STEC isolates differed in the type of Shiga toxin produced and originated from calves from different farms.

## 5 Conclusion

Our study maps the prevalence of different genotypes of ESBL-producing *E. coli* in calves from different dairy farms across multiple regions of the Czech Republic. Only five farms out of 23 sampled were culture-negative, highlighting that resistance to third-generation cephalosporins remains widespread, despite efforts to limit the use of these substances. Certain clonal lineages appear to have a higher colonization potential and are repeatedly found across different farms, where they can efficiently colonize farm care staff who have close contact with the animals.

## Data Availability

The data generated for this study can be found in the BioProject Database under https://www.ncbi.nlm.nih.gov/bioproject/1197128.

## Footnotes

^1^ https://github.com/tseemann/snp-dists

## References

[1] Gelalcha BD Gelgie AE Dego OK. Prevalence and antimicrobial resistance profiles of extended-spectrum beta-lactamase-producing *Escherichia coli* in East Tennessee dairy farms. Front Vet Sci. (2023) 10:1260433. doi: 10.3389/fvets.2023.1260433, PMID: 38239744 PMC10795760

[2] Weber LP Dreyer S Heppelmann M Schaufler K Homeier-Bachmann T Bachmann L. Prevalence and risk factors for ESBL/AmpC-*E. coli* in pre-weaned dairy calves on dairy farms in Germany. Microorganisms. (2021) 9:2135. doi: 10.3390/microorganisms9102135, PMID: 34683456 PMC8539614

[3] Gonggrijp MA Santman-Berends IMGA Heuvelink AE Buter GJ van Schaik G Hage JJ. Prevalence and risk factors for extended-spectrum β-lactamase- and AmpC-producing *Escherichia coli* in dairy farms. J Dairy Sci. (2016) 99:9001–13. doi: 10.3168/jds.2016-11134, PMID: 27638264

[4] Hille K Ruddat I Schmid A Hering J Hartmann M von Münchhausen C. Cefotaxime-resistant *E. coli* in dairy and beef cattle farms—joint analyses of two cross-sectional investigations in Germany. Prev Vet Med. (2017) 142:39–45. doi: 10.1016/j.prevetmed.2017.05.003, PMID: 28606364

[5] Homeier-Bachmann T Kleist JF Schütz AK Bachmann L. Distribution of ESBL/AmpC-*Escherichia coli* on a dairy farm. Antibiotics. (2022) 11:940. doi: 10.3390/antibiotics11070940, PMID: 35884193 PMC9311582

[6] Ludden C Raven KE Jamrozy D Gouliouris T Blane B Coll F. One health genomic surveillance of *Escherichia coli* demonstrates distinct lineages and mobile genetic elements in isolates from humans versus livestock. MBio. (2019) 10:e02693–18. doi: 10.1128/mBio.02693-18, PMID: 30670621 PMC6343043

[7] Dorado-García A Smid JH van Pelt W Bonten MJM Fluit AC van den Bunt G. Molecular relatedness of ESBL/AmpC-producing *Escherichia coli* from humans, animals, food and the environment: a pooled analysis. J Antimicrob Chemother. (2018) 73:339–47. doi: 10.1093/jac/dkx397, PMID: 29165596

[8] Iramiot JS Kajumbula H Bazira J Kansiime C Asiimwe BB. Antimicrobial resistance at the human–animal interface in the pastoralist communities of Kasese District, South Western Uganda. Sci Rep. (2020) 10:14737. doi: 10.1038/s41598-020-70517-w, PMID: 32895433 PMC7477235

[9] Qureshi MHF Azam F Shafique M Aslam B Farooq M Ur Rehman A. A one health perspective of pet birds bacterial zoonosis and prevention. Pak Vet J. (2024) 44:1–8. doi: 10.29261/pakvetj/2024.147

[10] Touchon M Perrin A de Sousa JAM Vangchhia B Burn S O'Brien CL. Phylogenetic background and habitat drive the genetic diversification of *Escherichia coli*. PLoS Genet. (2020) 16:e1008866. doi: 10.1371/journal.pgen.1008866, PMID: 32530914 PMC7314097

[11] Galarce N Sánchez F Escobar B Lapierre L Cornejo J Alegría-Morán R. Genomic epidemiology of Shiga toxin-producing *Escherichia coli* isolated from the livestock-food-human interface in South America. Animals. (2021) 11:1845. doi: 10.3390/ani11071845, PMID: 34206206 PMC8300192

[12] CLSI. Performance standards for antimicrobial susceptibility testing. Twenty-fifth informational supplement. CLSI document M100-S25. Wayne, PA: Clinical and Laboratory Standards Institute (2015).

[13] Masarikova M Sukkar I Jamborova I Medvecky M Papousek I Literak I. Antibiotic-resistant *Escherichia coli* from treated municipal wastewaters and black-headed Gull nestlings on the recipient river. One Health. (2024) 19:100901. doi: 10.1016/j.onehlt.2024.100901, PMID: 39399230 PMC11470789

[14] Bolger AM Lohse M Usadel B. Trimmomatic: a flexible trimmer for Illumina sequence data. Bioinformatics. (2014) 30:2114–20. doi: 10.1093/bioinformatics/btu170, PMID: 24695404 PMC4103590

[15] Bankevich A Nurk S Antipov D Gurevich AA Dvorkin M Kulikov AS. SPAdes: a new genome assembly algorithm and its applications to single-cell sequencing. J Comput Biol. (2012) 19:455–77. doi: 10.1089/cmb.2012.0021, PMID: 22506599 PMC3342519

[16] Bortolaia V Kaas RS Ruppe E Roberts MC Schwarz S Cattoir V. ResFinder 4.0 for predictions of phenotypes from genotypes. J Antimicrob Chemother. (2020) 75:3491–500. doi: 10.1093/jac/dkaa345, PMID: 32780112 PMC7662176

[17] Zankari E Hasman H Cosentino S Vestergaard M Rasmussen S Lund O. Identification of acquired antimicrobial resistance genes. J Antimicrob Chemother. (2012) 67:2640–4. doi: 10.1093/jac/dks261, PMID: 22782487 PMC3468078

[18] Carattoli A Zankari E Garciá-Fernández A Larsen MV Lund O Villa L. *In silico* detection and typing of plasmids using PlasmidFinder and plasmid multilocus sequence typing. Antimicrob Agents Chemother. (2014) 58:3895–903. doi: 10.1128/AAC.02412-14, PMID: 24777092 PMC4068535

[19] Liu B Zheng D Jin Q Chen L Yang J. VFDB 2019: a comparative pathogenomic platform with an interactive web interface. Nucleic Acids Res. (2019) 47:D687–92. doi: 10.1093/nar/gky1080, PMID: 30395255 PMC6324032

[20] Larsen MV Cosentino S Rasmussen S Friis C Hasman H Marvig RL. Multilocus sequence typing of total-genome-sequenced bacteria. J Clin Microbiol. (2012) 50:1355–61. doi: 10.1128/JCM.06094-11, PMID: 22238442 PMC3318499

[21] Joensen KG Tetzschner AM Iguchi A Aarestrup FM Scheutz F. Rapid and easy in silico serotyping of *Escherichia coli* using whole genome sequencing (WGS) data. J Clin Microbiol. (2015) 53:2410–26. doi: 10.1128/JCM.00008-15, PMID: 25972421 PMC4508402

[22] Beghain J. Bridier-Nahmias A. Nagard H. Le Denamur E. Clermont O. (2018). ClermonTyping: an easy-to-use and accurate in silico method for *Escherichia* genus strain phylotyping. Microb Genomics 4,:e000192. doi: 10.1099/mgen.0.000192, PMID: 29916797 PMC6113867

[23] Seemann T. Prokka: rapid prokaryotic genome annotation. Bioinformatics. (2014) 30:2068–9. doi: 10.1093/bioinformatics/btu153, PMID: 24642063

[24] Bayliss SC Thorpe HA Coyle NM Sheppard SK Feil EJ. PIRATE: a fast and scalable pangenomics toolbox for clustering diverged orthologues in bacteria. Gigascience. (2019) 8:giz119. doi: 10.1093/gigascience/giz119, PMID: 31598686 PMC6785682

[25] Stamatakis A. RAxML version 8: a tool for phylogenetic analysis and post-analysis of large phylogenies. Bioinformatics. (2014) 30:1312–3. doi: 10.1093/bioinformatics/btu033, PMID: 24451623 PMC3998144

[26] Letunic I Bork P. Interactive tree of life (iTOL) v3: an online tool for the display and annotation of phylogenetic and other trees. Nucleic Acids Res. (2016) 44:W242–5. doi: 10.1093/NAR/GKW290, PMID: 27095192 PMC4987883

[27] Letunic I Bork P. Interactive tree of life (iTOL) v6: recent updates to the phylogenetic tree display and annotation tool. Nucleic Acids Res. (2024) 52:W78–82. doi: 10.1093/nar/gkae268, PMID: 38613393 PMC11223838

[28] Arikan MS Mat B Alkan H Çevrimli MB Akin AC Başar EK. Determination of subclinical mastitis prevalence in dairy cows in Türkiye through MetaAnalysis and production loss calculation. Pak Vet J. (2024) 44:391–9. doi: 10.29261/pakvetj/2024.142, PMID: 39902308 PMC11787863

[29] Tello M Ocejo M Oporto B Lavín JL Hurtado A. Within-farm dynamics of ESBL-producing *Escherichia coli* in dairy cattle: resistance profiles and molecular characterization by long-read whole-genome sequencing. Front Microbiol. (2022) 13:936843. doi: 10.3389/fmicb.2022.936843, PMID: 35966684 PMC9366117

[30] Zamudio R Boerlin P Beyrouthy R Madec JY Schwarz S Mulvey MR. Dynamics of extended-spectrum cephalosporin resistance genes in *Escherichia coli* from Europe and North America. Nat Commun. (2022) 13:7490. doi: 10.1038/s41467-022-34970-7, PMID: 36509735 PMC9744880

[31] Manga I Hasman H Smidkova J Medvecky M Dolejska M Cizek A. Fecal carriage and whole-genome sequencing-assisted characterization of CMY-2 beta-lactamase-producing *Escherichia coli* in calves at Czech dairy cow farm. Foodborne Pathog Dis. (2019) 16:42–53. doi: 10.1089/fpd.2018.2531, PMID: 30673354

[32] Li Y Ma X Li C Dai X Zhang L. Occurrence and genomic characterization of ESBL-producing *Escherichia coli* ST29 strains from swine with abundant virulence genes. Microb Pathog. (2020) 148:104483. doi: 10.1016/j.micpath.2020.104483, PMID: 32918980

[33] Massot M Châtre P Condamine B Métayer V Clermont O Madec J-Y. Interplay between bacterial clones and plasmids in the spread of antibiotic resistance genes in the gut: lessons from a temporal study in veal calves. Appl Environ Microbiol. (2021) 87:e0135821. doi: 10.1128/AEM.01358-21, PMID: 34613750 PMC8612258

[34] Valiakos G Kapna I. Colistin resistant *mcr* genes prevalence in livestock animals (swine, bovine, poultry) from a multinational perspective a systematic review. Vet Sci. (2021) 8:265. doi: 10.3390/vetsci8110265, PMID: 34822638 PMC8619609

[35] Massé J Vanier G Fairbrother JM de Lagarde M Arsenault J Francoz D. Description of antimicrobial-resistant *Escherichia coli* and their dissemination mechanisms on dairy farms. Vet Sci. (2023) 10:242. doi: 10.3390/vetsci10040242, PMID: 37104397 PMC10144642

[36] Arimizu Y Kirino Y Sato MP Uno K Sato T Gotoh Y. Large-scale genome analysis of bovine commensal *Escherichia coli* reveals that bovine-adapted *E. coli* lineages are serving as evolutionary sources of the emergence of human intestinal pathogenic strains. Genome Res. (2019) 29:1495–505. doi: 10.1101/gr.249268.119, PMID: 31439690 PMC6724679

[37] Massella E Giacometti F Bonilauri P Reid CJ Djordjevic SP Merialdi G. Antimicrobial resistance profile and ExPEC virulence potential in commensal *Escherichia coli* of multiple sources. Antibiotics. (2021) 10:351. doi: 10.3390/antibiotics10040351, PMID: 33810387 PMC8067153

[38] He WY Zhang XX Gao GL Gao MY Zhong FG Lv LC. Clonal spread of *Escherichia coli* O101:H9-ST10 and O101:H9-ST167 strains carrying *fosA3* and *bla*_CTX-M_-_14_ among diarrheal calves in a Chinese farm, with Australian *Chroicocephalus* as the possible origin of *E. coli* O101:H9-ST10. Zool Res. (2021) 42:461–8. doi: 10.24272/J.ISSN.2095-8137.2021.153 34156173 PMC8317193

[39] Gonzalez-Escalona N Toro M Rump LV Cao G Nagaraja TG Meng J. Virulence gene profiles and clonal relationships of *Escherichia coli* O26:H11 isolates from feedlot cattle as determined by whole-genome sequencing. Appl Environ Microbiol. (2016) 82:3900–12. doi: 10.1128/AEM.00498-16, PMID: 27107118 PMC4907181

[40] Irrgang A Roschanski N Tenhagen B-A Grobbel M Skladnikiewicz-Ziemer T Thomas K. Prevalence of mcr-1 in E. coli from Livestock and Food in Germany, 2010–2015. PLoS ONE. (2016) 11:e0159863. doi: 10.1371/journal.pone.0159863 27454527 PMC4959773

[41] Sano E Esposito F Fontana H Fuga B Cardenas-Arias A Moura Q One health clones of multidrug-resistant Escherichia coli carried by synanthropic animals in Brazil. One Health. (2022) 16:100476. doi: 10.1016/j.onehlt.2022.100476 36691392 PMC9860340

