# Supplementary material for: Genomic analysis of extended-spectrum beta-lactamase-producing E. coli from Czech diary calves and their caretakers
Source: Front Vet Sci. 2025 Mar 12;12:1552297. doi: 10.3389/fvets.2025.1552297 (PMC11938843; doi:10.3389/fvets.2025.1552297)
Supplement: Supplementary file 1 [file Table_1.DOCX]

**Table S1: Overview of farms, processed samples, isolated and sequenced *E. coli*.**

| Farm | Place of collection | No. of processed samples | Culture positive (MCA_cef_) samples | No. of sequenced isolates |
| --- | --- | --- | --- | --- |
| A | *CF | 16 | 9 | 8 |
| B | *CF | 2 | 0 | 0 |
| C | *CF | 4 | 4 | 4 |
| D | *CF | 3 | 2 | 2 |
| E | *CF | 6 | 6 | 5 |
| F | *CF | 2 | 1 | 1 |
| G | *CF | 2 | 2 | 0 |
| H | *CF | 11 | 11 | 6 |
| I | *CF | 14 | 14 | 7 |
| J | *CF | 10 | 6 | 5 |
| K | *CF | 7 | 0 | 0 |
| L | *CF | 2 | 0 | 0 |
| M | *CF | 11 | 1 | 1 |
| N | *CF | 5 | 0 | 0 |
| O | *CF | 16 | 13 | 6 |
| P | *CF | 21 | 14 | 11 |
| Q | *CF | 8 | 8 | 3 |
| R | *CF | 44 | 0 | 0 |
| S | S | 20 | 16 | 5 |
| T | T | 29 | 2 | 2 |
| U | U | 20 | 7 | 1 |
| V | V | 12 | 11 | 1 |
| W | *CF | 1 | 1 | 0 |
|  | **Total** | **266** | **128** | **68** |
| *CF | ***CF - 3 caretakers and 1 vet** | **4** | **3 (all caretakers)** | **15** |

***** Collection Farm.
