# Supplementary material for: Genomic analysis of extended-spectrum beta-lactamase-producing E. coli from Czech diary calves and their caretakers
Source: Front Vet Sci. 2025 Mar 12;12:1552297. doi: 10.3389/fvets.2025.1552297 (PMC11938843; doi:10.3389/fvets.2025.1552297)
Supplement: Supplementary file 2 [file Table_2.DOCX]

**Table S2:** **Resistance phenotype and genotype of 128 calf and 15 human isolates.**

| Isolate | Origin | Farm | Resistance phenotype | Resistance genotype |
| --- | --- | --- | --- | --- |
| 60 | calf | A | Amp, Cf, Caz, S, Su, T | *bla*_TEM_, *bla*_CTX-M_, *qnrS* |
| 62 | calf | A | Amp, Cf, Caz, Fox, S, Su, T | *bla*_TEM_, *bla*_CTX-M_, *qnrS* |
| 64 | calf | A | Amp, Cf, Caz, Fox, S, Su, T | *bla*_TEM_, *bla*_CTX-M_, *qnrS* |
| 66 | calf | A | Amp, Cf, Caz, Fox, S, Su, T | *bla*_TEM_, *bla*_CTX-M_, *qnrS* |
| 67 | calf | A | Amp, Cf, S, Su, T | *bla*_TEM_, *bla*_CTX-M_, *qnrS* |
| 68 | calf | A | Amp, Cf, Caz, Fox, S, Su, T | *bla*_TEM_, *bla*_CTX-M_, *qnrS* |
| 70 | calf | A | Amp, Cf, Caz, Fox, S, Su, T | *bla*_TEM_, *bla*_CTX-M_, *qnrS* |
| 49 | calf | A | Amp, Cf, Caz, S, Su | *bla*_TEM_, *bla*_CTX-M_, *qnrS* |
| 50 | calf | A | Amp, Amc, Cf, C, Fox, S, Su, Sxt,T | - |
| 53 | calf | C | Amp, Amc, Cf, C, Fox, Gn, S, Su, Sxt,T | *bla*_CTX-M_ |
| 54 | calf | C | Amp, Cf, C, Fox, Su | *bla*_CTX-M_ |
| 55 | calf | C | Amp, Amc, Cf, C, Fox, Gn, S, Su, Sxt,T | *bla*_CTX-M_ |
| 56 | calf | C | Amp, Cf | *bla*_CTX-M_ |
| 71 | calf | D | Amp, Cf, Fox, Su, T | *bla*_CTX-M_ |
| 94 | calf | D | Amp, Cf, Fox, Su, T | *bla*_CTX-M_ |
| 73 | calf | E | Amp, Amc, Cf, Fox, S, T | *bla*_TEM_, *bla*_CTX-M_ |
| 74 | calf | E | Amp, Cf, C, Fox, S, Su, T | *bla*_CTX-M_ |
| 75 | calf | E | Amp, Cf, Fox, S, Su | *bla*_TEM_, *bla*_CTX-M_ |
| 76 | calf | E | Amp, Cf, Fox, S, Su, T | *bla*_TEM_, *bla*_CTX-M_ |
| 77 | calf | E | Amp, Amc, Cf, S, Su, T | *bla*_TEM_, *bla*_CTX-M_ |
| 78 | calf | E | Amp, Cf, S, Su, T | *bla*_TEM_, *bla*_CTX-M_ |
| 79 | calf | F | Amp, Amc, Cf, C, Fox, S, Su, Sxt, T | *bla*_TEM_, *bla*_CTX-M_ |
| 81 | calf | G | Amp, Cf, S, Su, Sxt, T | *bla*_TEM_, *bla*_CTX-M_ |
| 82 | calf | G | Amp, Amc, Cf, Fox, S, Su, Sxt, T | *bla*_TEM_, *bla*_CTX-M_ |
| 83 | calf | H | Amp, Cf, Fox, T | *bla*_CTX-M_ |
| 84 | calf | H | Amp, Cf, C, Fox, S, Su, Sxt, T | *bla*_TEM_, *bla*_CTX-M_ |
| 85 | calf | H | Amp, Cf, C, S, Su, Sxt, T | *bla*_TEM_, *bla*_CTX-M_ |
| 86 | calf | H | Amp, Amc, Cf, C, Fox, S, Su, T | *bla*_CTX-M_ |
| 87 | calf | H | Amp, Cf, Fox, T | *bla*_CTX-M_ |
| 88 | calf | H | Amp, Cf, C, Fox, S, Su, Sxt, T | *bla*_TEM_, *bla*_CTX-M_ |
| 89 | calf | H | Amp, Cf, Fox | *bla*_CTX-M_ |
| 90 | calf | H | Amp, Cf, C, Fox, S, Su, T | *bla*_CTX-M_ |
| 91 | calf | H | Amp, Cf, C, Fox, S, Su, T | *bla*_CTX-M_ |
| 92 | calf | H | Amp, Cf, Fox, S, T | *bla*_CTX-M_ |
| 93 | calf | H | Amp, Cf, C, Fox, S, Su, T | *bla*_CTX-M_ |
| 104 | calf | I | Amp, Cf, C, Cip, Fox, Na, S, Su, Sxt, T | *bla*_TEM_, *bla*_CTX-M_ |
| 105 | calf | I | Amp, Cf, C, Cip, Na, S, Su, Sxt, T | *bla*_TEM_, *bla*_CTX-M_ |
| 106 | calf | I | Amp, Fox, S, T | *bla*_TEM_ |
| 107 | calf | I | Amp, Cf, C, Cip, Na, S, Su, Sxt, T | *bla*_TEM_, *bla*_CTX-M_ |
| 108 | calf | I | Amp, Cf, Na, S, Su, T | *bla*_TEM_, *bla*_CTX-M_ |
| 109 | calf | I | Amp, Amc, Cf, C, Cip, Na, S, Su, Sxt, T | *bla*_TEM_, *bla*_CTX-M_ |
| 110 | calf | I | Amp, Cf, S, Su, T | *bla*_TEM_, *bla*_CTX-M_ |
| 113 | calf | I | Amp, Cf, S, Su, Sxt, T | - |
| 114 | calf | I | Amp, Cf, S, Su, T | *bla*_TEM_, *bla*_CTX-M_ |
| 115 | calf | I | Amp, Amc, Cf, S, Su, T | *bla*_TEM_, *bla*_CTX-M_ |
| 116 | calf | I | Amp, Amc, Cf, S, Su, T | *bla*_TEM_, *bla*_CTX-M_ |
| 117 | calf | I | Amp, Amc, Cf, S, Su, T | *bla*_TEM_, *bla*_CTX-M_ |
| 118 | calf | I | Amp, Cf, Na, S, Su, T | *bla*_TEM_, *bla*_CTX-M_ |
| 119 | calf | I | Amp, Cf, S, Su, T | *bla*_TEM_, *bla*_CTX-M_ |
| 189 | calf | J | Amp, Amc, Cf, Cip, Fox, Na, S, Su, Sxt, T | *bla*_CTX-M_ |
| 190 | calf | J | Amp, Amc, Cf, C, Cip, Fox, Gn, Na, S, Su, Sxt, T | *bla*_CTX-M_ |
| 191 | calf | J | Amp, Amc, Cf, C, Su, Sxt, T | *bla*_TEM_ |
| 193 | calf | J | Amp, Amc, Cf, S, Su, Sxt, T | *bla*_TEM_ |
| 111 | calf | J | Amp, Cf, C, Cip, Gn, Na, S, Su, Sxt, T | *bla*_TEM_ |
| 112 | calf | J | Amp, Cf, S, Su, T | *bla*_TEM_, *bla*_CTX-M_ |
| 136 | calf | M | Amp, Amc, Cf, Fox | - |
| 148 | calf | O | Amp, Cf | *bla*_CTX-M_ |
| 149 | calf | O | Amp, Amc, Cf, S, Su, Sxt, T | *bla*_TEM_, *bla*_CTX-M_ |
| 150 | calf | O | Amp, Amc, Cf, C, Su, T | *bla*_CTX-M_ |
| 151 | calf | O | Amp, Cf, S, Su, Sxt, T | *bla*_TEM_, *bla*_CTX-M_ |
| 152 | calf | O | Amp, Cf | *bla*_CTX-M_ |
| 153 | calf | O | Amp, Amc, Cf, S, Su, Sxt, T | *bla*_TEM_, *bla*_CTX-M_ |
| 155 | calf | O | Amp, Cf | *bla*_CTX-M_ |
| 156 | calf | O | Amp, Amc, Cf | *bla*_CTX-M_ |
| 158 | calf | O | Amp, Amc, Cf, Caz | *bla*_CTX-M_ |
| 159 | calf | O | Amp, Amc, Cf | *bla*_CTX-M_ |
| 161 | calf | O | Amp, Cf | *bla*_CTX-M_ |
| 162 | calf | O | Amp, Cf | *bla*_CTX-M_ |
| 163 | calf | O | Amp, Amc, Cf | *bla*_CTX-M_ |
| 168 | calf | P | Amp, Amc, Cf, Cip, Na, S, Su, T | *bla*_CTX-M_ |
| 171 | calf | P | Amp, Amc, Cf, C, Cip, Gn, Na, S, Su, Sxt, T | *bla*_CTX-M_ |
| 172 | calf | P | Amp, Amc, Cf, C, Cip, Gn, Na, S, Su, Sxt, T | *bla*_CTX-M_ |
| 174 | calf | P | Amp, Amc, Cf, C, Cip, Fox, Gn, Na, S, Su, Sxt, T | *bla*_CTX-M_ |
| 175 | calf | P | Amp, Amc, Cf, C, Su | *bla*_CTX-M_ |
| 176 | calf | P | Amp, Amc, Cf, C, Cip, Gn, Na, S, Su, Sxt, T | *bla*_CTX-M_ |
| 177 | calf | P | Amp, Amc, Cf, C, Cip, Gn, Na, S, Su, Sxt, T | *bla*_CTX-M_ |
| 181 | calf | P | Amp, Amc, Cf, C, Cip, Gn, Na, S, Su, Sxt, T | *bla*_CTX-M_ |
| 182 | calf | P | Amp, Amc, Cf, C, Cip, Fox, Gn, Na, Su, T | *bla*_TEM_, *bla*_CTX-M_ |
| 184 | calf | P | Amp, Amc, Cf, C, Cip, Gn, Na, S, Su, Sxt, T | *bla*_CTX-M_ |
| 185 | calf | P | Amp, Amc, Cf, Caz, C, Fox, S, Su, T | - |
| 186 | calf | P | Amp, Amc, Cf, C, Cip, Fox, Gn, Na, S, Su, Sxt, T | *bla*_CTX-M_ |
| 187 | calf | P | Amp, Amc, Cf, C, Cip, Fox, Gn, Na, Su, T | *bla*_TEM_, *bla*_CTX-M_ |
| 188 | calf | P | Amp, Amc, Cf, C, Fox, S, Su, Sxt, T | *bla*_TEM_, *qnrS* |
| 96 | calf | Q | Amp, Amc, Cf, C, Cip, Na, S, Su, Sxt, T | *bla*_TEM_, *bla*_CTX-M_ |
| 97 | calf | Q | Amp, Amc, Cf, Caz, Fox, S, T | - |
| 98 | calf | Q | Amp, Amc, Cf, Caz, Fox, S | - |
| 99 | calf | Q | Amp, Amc, Cf, Caz, Fox, S, T | - |
| 100 | calf | Q | Amp, Cf, C, Cip, Fox, Gn, Na, S, Su, Sxt, T | *bla*_CTX-M_ |
| 101 | calf | Q | Amp, Amc, Cf, Caz, Fox, S, T | - |
| 102 | calf | Q | Amp, Cf, C, Cip, Fox, Gn, Na, S, Su, Sxt, T | *bla*_CTX-M_ |
| 103 | calf | Q | Amp, Cf, C, Cip, Fox, Gn, Na, S, Su, Sxt, T | *bla*_CTX-M_ |
| 236 | calf | S | Amp, Amc, Cf, S, Su, Sxt, T | *bla*_CTX-M_ |
| 237 | calf | S | Amp, Amc, Cf, Caz | *bla*_CTX-M_ |
| 238 | calf | S | Amp, Amc, Cf | *bla*_CTX-M_ |
| 239 | calf | S | Amp, Cf, Caz | *bla*_CTX-M_ |
| 241 | calf | S | Amp, Cf, Caz | *bla*_CTX-M_ |
| 242 | calf | S | Amp, Cf, Caz | *bla*_CTX-M_ |
| 243 | calf | S | Amp, Cf, Caz | *bla*_CTX-M_ |
| 244 | calf | S | Amp, Cf, Caz | *bla*_CTX-M_ |
| 245 | calf | S | Amp, Cf, Caz | *bla*_CTX-M_ |
| 248 | calf | S | Amp, Cf, Caz | *bla*_CTX-M_ |
| 249 | calf | S | Amp, Cf | *bla*_CTX-M_ |
| 250 | calf | S | Amp, Cf | *bla*_CTX-M_ |
| 251 | calf | S | Amp, Cf | *bla*_CTX-M_ |
| 252 | calf | S | Amp, Cf | *bla*_CTX-M_ |
| 253 | calf | S | Amp, Cf | *bla*_CTX-M_ |
| 255 | calf | S | Amp, Cf | *bla*_CTX-M_ |
| 266 | calf | T | Amp, Cf | *bla*_CTX-M_ |
| 274 | calf | T | Amp, Cf, Caz, S, Su, Sxt, T | *bla*_TEM_, *bla*_CTX-M_, *qnrS* |
| 316 | calf | U | Amp, Amc, Cf, Caz, C, S, T | *bla*_TEM_, *bla*_CTX-M_, *qnrS* |
| 323 | calf | U | Amp, Cf, S, Su, Sxt, T | *bla*_TEM_, *bla*_CTX-M_ |
| 321 | calf | U | Amp, Cf, Caz, S, T | *bla*_TEM_, *bla*_CTX-M_, *qnrS* |
| 320 | calf | U | Amp, Cf, C, S, Su, Sxt, T | *bla*_TEM_, *bla*_CTX-M_ |
| 319 | calf | U | Amp, Cf, Caz, C, S, T | *bla*_TEM_, *bla*_CTX-M_, *qnrS* |
| 318 | calf | U | Amp, Amc, Cf, Caz, C, S, T | *bla*_TEM_, *bla*_CTX-M_, *qnrS* |
| 313 | calf | U | Amp, Amc, Cf, Caz, C, S, T | *bla*_TEM_, *bla*_CTX-M_, *qnrS* |
| 331 | calf | V | Amp, Amc, Cf, Caz, C, S, T | *bla*_TEM_, *bla*_CTX-M_, *qnrS* |
| 332 | calf | V | Amp, Cf, Caz, C, S, T | *bla*_TEM_, *bla*_CTX-M_, *qnrS* |
| 333 | calf | V | Amp, Cf, Caz, C, S, T | *bla*_TEM_, *bla*_CTX-M_, *qnrS* |
| 335 | calf | V | Amp, Cf, Caz, C, S, T | *bla*_TEM_, *bla*_CTX-M_, *qnrS* |
| 336 | calf | V | Amp, Cf, Caz, C, S, T | *bla*_TEM_, *bla*_CTX-M_, *qnrS* |
| 337 | calf | V | Amp, Cf, Caz, C, S, T | *bla*_TEM_, *bla*_CTX-M_, *qnrS* |
| 338 | calf | V | Amp, Cf, Caz, C, S, T | *bla*_TEM_, *bla*_CTX-M_, *qnrS* |
| 339 | calf | V | Amp, Cf, Caz, C, S, T | *bla*_TEM_, *bla*_CTX-M_, *qnrS* |
| 340 | calf | V | Amp, Amc, Cf, Caz, C, S, T | *bla*_TEM_, *bla*_CTX-M_, *qnrS* |
| 341 | calf | V | Amp, Cf, Caz, C, S, Su, T | *bla*_TEM_, *bla*_CTX-M_, *qnrS* |
| 342 | calf | V | Amp, Cf, Caz, C, S, T | *bla*_TEM_, *bla*_CTX-M_, *qnrS* |
| 95 | calf | W | Amp, Cf, Caz, Fox, S, Su, T | *bla*_TEM_, *bla*_CTX-M_, *qnrS* |
| Os K e1 | human | *CF | Amp, Amc, C, Cf, Cip, Ctx, Gn, Na, S, Su, Sxt, T | *bla*_CTX-M_ |
| Os K e2 | human | *CF | Amp, Amc, C, Cf, Cip, Ctx, Gn, Na, S, Su, Sxt, T | *bla*_TEM_, *bla*_CTX-M_ |
| Os K e3 | human | *CF | Amp, C, Cf, Cip, Ctx, Gn, Na, S, Su, Sxt, T | *bla*_CTX-M_ |
| Os K e4 | human | *CF | Amp, C, Cf, Cip, Ctx, Gn, Na, S, Su, Sxt, T | *bla*_TEM_, *bla*_CTX-M_ |
| Os K e5 | human | *CF | Amp, Amc, C, Cf, Cip, Ctx, Gn, Na, S, Su, Sxt, T | *bla*_TEM_, *bla*_CTX-M_ |
| Os L e1 | human | *CF | Amp, Amc, C, Cf, S, Su, Sxt, T | *bla*_TEM_ |
| Os L e2 | human | *CF | Amp, C, Cf, S, Su, Sxt, T | *bla*_TEM_ |
| Os L e3 | human | *CF | Amp, Amc, C, Cf, Ctx, Gn, Na, S, Su, Sxt, T | *bla*_CTX-M_ |
| Os L e4 | human | *CF | Amp, C, Cf, S, Su, Sxt, T | *bla*_TEM_ |
| Os L e5 | human | *CF | Amp, Amc, C, Cf, S, Su, Sxt, T | *bla*_TEM_ |
| Os S e1 | human | *CF | Amp, Amc, C, Cf, S, Su, Sxt, T | *bla*_TEM_ |
| Os S e2 | human | *CF | Amp, Amc, C, Cf, S, Su, Sxt, T | *bla*_TEM_ |
| Os S e3 | human | *CF | Amp, C, Cf, S, Su, Sxt, T | *bla*_TEM_ |
| Os S e4 | human | *CF | Amp, Amc, C, Cf, S, Su, Sxt, T | *bla*_TEM_ |
| Os S e5 | human | *CF | Amp, C, Cf, S, Su, Sxt, T | *bla*_TEM_ |

*Collection Farm

Resistance phenotype: Amc: Amoxicillin-clavulanic acid, Amp: Ampicillin, Cf: Cephalotin, Fox: Cefoxitin, Caz: Ceftazidime, C: Chloramphenicol, Cip: Ciprofloxacin, Gn: Gentamicin, Na: Nalidixic acid, S: Streptomycin, Sxt: Sulfamethoxazole-trimethoprim, Su: Sulfonamides compound, T: Tetracycline.
